# Facilitating factors and barriers to accessibility and utilization of kangaroo mother care service among parents of low birth weight infants in Mangochi District, Malawi: a qualitative study

**DOI:** 10.1186/s12887-020-02251-1

**Published:** 2020-07-29

**Authors:** Christina T. Mathias, Solange Mianda, Themba G. Ginindza

**Affiliations:** 1 Discipline of Public Health Medicine, School of Nursing and Public Health, College of Health Science, University of KwaZulu-Natal, 2nd Floor George Campbell Building, Mazisi Kunene Road, Durban, 4041 South Africa; 2 School of Public Health, University of the Western Cape, Cape Town, South Africa

**Keywords:** Barriers, Challenges, Experience, Kangaroo mother care and facilitating factors

## Abstract

**Background:**

Kangaroo Mother Care (KMC) is one of the interventions widely used in low-income countries to manage Low Birth Weight Infants (LBWIs), a global leading cause of neonatal and child mortality. LBWI largely contributes to neonatal mortality in Malawi despite the country strengthening and implementing KMC, nationwide, to enhance the survival of LBWIs. This qualitative study aimed to assess the facilitating factors and barriers to accessibility and utilization of KMC service by the parent of low birth weight infants (PLBWIs) in Mangochi District, Malawi.

**Methods:**

Two focused group discussions assessed factors facilitating and hindering the accessibility and utilization of KMC service were conducted in April 2018 that reached out to (*N* = 12) participants; (n:6) PLBWI practicing KMC at Mangochi district hospital (MDH) referred from four health facilities and (n:6) high-risk pregnant mothers (HRPMs) visiting antenatal care (ANC) clinic at MDH. The availability of KMC at MDH was assessed using KMC availability checklist. The study used purposive, convenient and simple random sampling to identify eligible participants. Thematic analysis was used to analyze the findings.

**Results:**

Sixteen themes emerged on facilitating factors and barriers to accessibility and utilization of KMC service by the PLBWIs. The identified themes included; availability of KMC providers, social factor (social support and maternal love), timing of KMC information, knowledge on KMC, health linkage systems, recognition of LBWIs, safety on the use of KMC, preference of LBWI’s care practice, lived experience on KMC practice, KMC expert clients, perceived causes of LBWI births, cultural/traditional factors, religious beliefs, health-seeking behavior, women empowerment and quality of care.

**Conclusions:**

Although KMC was available in some of the health facilities, integration of KMC messages in ANC guidelines, community awareness and in sensitization of any health intervention may enhance KMC accessibility and utilization by the targeted population.

## Background

Kangaroo Mother Care (KMC), a skin-to-skin contact approach between the low birth weight infant [LBWI] [1, 2] enhances LBWIs survival by more than 50% [3–6]. Annually, 18 million LBWIs are born in Low and Middle-Income Countries [LMICs] that accounts for 90% of the LBWIs born globally [7]. LBWIs’ mortality is higher in developing countries than in developed countries, which accounts for 60–80% of the global neonatal mortality [7, 8]. This evidently shows that LBWIs’ mortality contributes largely to the global neonatal deaths. Annually, more than 9 million LBWIs born in LMICs die due to low social-economic status and poor health-seeking behavior [1, 2]. LMICs mostly use KMC intervention due to its low-cost effective measures and for its numerous empirical evident medical benefits on the LBWIs [3–6].

Malawi strengthens the implementation of KMC by integrating KMC approach in national health care initiatives and in medical and nursing qualification training [3–5], which has facilitated the implementation of KMC service in almost 88.5% of the national health facilities [6, 9]. Despite the national initiatives on KMC and wider implementation of the service, LBWI is still a national leading cause of neonatal mortality [8, 10], with Mangochi district registering a high prevalence of LBWIs and neonatal mortality of 40 per 1000 live birth [11, 12]. This denotes that KMC utilization is equally a challenge in Mangochi district despite most of the health facilities provide KMC service to mitigate LBWIs complications.

The involvement of the stakeholders including the beneficiaries of the service is paramount in the utilization and success of the intervention [13, 14], as optimal, quality and desirable outcomes of care depend on the key providers and beneficiaries [15]. Therefore, the measure of the success of KMC service depends on the access and utilization of the service by the beneficiaries, parents with LBWI [PLBWIs] and high-risk pregnant mothers [HRPMs], who are at risk of giving birth to LBWIs [14, 15]. Literature defines access as the availability, affordability, accessibility and acceptability of a service [14, 16, 17]. The utilization of the service by the beneficiaries mostly depends on access and the absence of challenges and barriers, perceived quality of care, cost of care, supportive factors, cultural factors, religious/cultural factors, health systems factors and provider factors [18–20]. As such, finding the empirical strategies on the issues facilitating the accessibility and utilization of KMC service, by the parents of LBWIs (PLBWIs, would facilitate the integration of strategies in LMICs that would effectively enhance the utilization and impact of KMC service on LBWIs lives. Unfortunately, there are fewer studies conducted focusing on the accessibility and utilization of the PLBWIs than studies focusing on KMC implementation [21]. Therefore, this study focuses on assessing the facilitating factors and barriers that facilitate accessibility and utilization of KMC service by PLBWIs in Mangochi district, Southern Malawi, to find recommendations/strategies/approaches to incorporate in the implementation of KMC service that will benefit the LBWIs. The findings will also inform future research and KMC policy/guidelines updates.

## Methods

### Aim of the study, study design and site

The study aimed to assess the facilitating factors and barriers to accessibility and utilization of KMC service by PLBWIs. The study used the cross-sectional design applying the qualitative approach. Two focus group discussions (FGDs) conducted that involved PLBWIs practicing KMC, and high-risk pregnant mothers (HRPMs) who had conditions predisposing them to preterm birth. The facility observation, participants’ recruitment and the study took place at Mangochi District Hospital (MDH), which offers maternity services including KMC service. All interviews,.conducted in organised private room for PLBWIs’ privacy in all the service delivery points.

### Procedures and characteristics of the participants

The study involved 12 participants (six PLBWIs and six HRPMs) whose socio-demographic characteristics shown in Table 1. The PLBWIs had six LBWIs whose demographic characteristics presented in Table 2. Although the sample size of the qualitative study is not defined, the sample size of this study provided the in-depth understanding of the phenomenon relevant to the phenomenon under investigation [22]. Asides the PLBWIs and the HRPMs, the study looked at the characteristics of the seven LBWIs born from the six PLBWIs as shown in Table 2. The researcher (investigator), a trained qualitative studies researcher, conducted two FGDs with six PLBWIs and six HRPMs in each FGD session, each lasting 1 h 30 min. Focused group sessions, using the FGD guides (see Additional files 1 and 2), conducted until no new themes identified from the discussions.

**Table 1:** Socio-demographic characteristics of study population (*N* = 12)

Demographics	n (%)
**Age (years)**	
**Mean ± SD (range)**	23.2 ± 8.2
15–19	6 (50.0)
20–24	2 (16.7)
25–29	2 (16.7)
> 30	2 (16.7)
**Marital status**	
Single	1 (8.3)
Married	11 (91.7)
**Education**	
Never been to school	1 (8.3)
Some primary school	1 (8.3)
Incomplete primary school	9 (75.1)
Complete secondary school	1 (8.3)
**Occupation**	
Unemployed	11 (91.7)
Employed	1 (8.3)
**Pregnant before**	
Yes	7 (58.3)
No	5 (41.7)
**Number of pregnancies**	
1	5 (41.7)
2	1 (8.3)
3	2 (16.7)
4+	4 (33.3)
**Predisposing factor to LBWI delivery**	
HIV, primigravida and adolescent	1 (8.3)
High Blood Pressure	1 (8.3)
HIV	4 (33.3)
Primigravida and adolescent	4 (33.3)
STI	1 (8.3)
Twin Gestation	1 (8.3)

**Table 2:** Demographic characteristics of Low Birth Weight Infants (*N* = 7)

Demographics	n (%)
**Gestational age (weeks)**	
**Mean gestational age ± SD (range)**	34.2 ± 1.5
30–32	1 (14.3)
33–35	5 (41.7)
36	1 (14.3)
**Birth weight (g)**	
**Mean birth weight ± SD (range)**	1958.3 ± 441.3
1000–1449	1 (14.3)
1500–1999	5 (71.4)
2000–2499	1 (14.3)
**Sex**	
Female	4 (57.1)
Male	3 (42.9)
**Attributing factors to LBWI delivery**	
Primigravida	4 (42.9)
Twin gestation	2 (28.5)
HIV	1 (14.3)

The PLBWIs practicing KMC were purposively selected as the researcher recruited participants that were assumed to have knowledge and experience on KMC and they shared the same characterizes as the phenomenon of the study was concerned. To ensure that all the PLBWIs had equal chances of participating in the study, a simple random sampling applied on the names, in the KMC register, of the LBWIs admitted in the MDH’s KMC unit. The selection of HRPMS purposively done at ANC clinic, in which typical case purposive sampling was used to recruit pregnant mothers who had similar characteristics to the PLBWIs. The HRPMs who had conditions such as HIV, anaemia, hypertension, diabetes, malaria and sexually transmitted infections (STIs) predisposing them to deliver preterm infants were conveniently approached by checking in their health passbooks. Table 1 shows the predisposing conditions to LBWIs deliveries by the PLBWIs and the HRPMs who participated in this study.

The identified participants approached and provided with the information sheet on the aim of the study, risk of the study, inclusion and exclusion criteria Participants who agreed to participate in the study read and signed a consent form.

The FGDs was guided by the structured interview questions (Additional files 1, 2 and 3) informed by the literature review, to address the aim of the study. The topics covered ranged from availability, accessibility, acceptability, affordability of KMC service, personal behavior and quality of care.

Confidentiality and anonymity of the participants maintained throughout the study, by assigning pseudo names to participants. The pseudo names contained a prefix and a suffix, where the prefix was the category group and the suffix was a number, for example, *HRPM 1.* The data were analyzed using a thematic analysis approach to code and analyze data [23]. Discussions conducted in local languages (Chichewa and ChiYao), upon transcription of the recordings and notes, the themes were identified and grouped depending on the similarities and differences. Grouped themes coded; guided by the study outcomes and emerging themes. The descriptive themes assigned to the coded themes to give them a descriptive meaning, which became the study findings.

## Results

Sixteen themes on facilitating factors and challenges that affect the accessibility and utilization of KMC service by the PLBWIs fell under availability, accessibility, acceptability, affordability of KMC service, personal behavior and quality of care, as presented in Table 3.

**Table 3:** Matrix of facilitating factors and barriers affecting the accessibility and utilization of KMC service by the PLBWIs in MDH, in 2018

	Availability of KMC service	Accessibility of KMC service	Acceptability of KMC service	Affordability of KMC service	Personal Behavior	Quality of care
**Facilitating factors**	Availability of KMC providers	Social support -Parents utilized KMC service 24 h	LBWI recognized as a human being	KMC perceived as a cheap service		
		Knowledge on KMC and timing of KMC message dissemination	KMC perceived as a safe service to an LBWI			
		Health linkage systems -refer LBWIs to secondary facility level for further management	Preference of KMC as LBWI care			
			Parental affection			
			Lived experience -positive outcome with KMC service			
			Motivation talks by mothers who practiced KMC and experience a positive outcome			
			Perceived causes of LBWI birth -medical and trauma			
**Barriers**	Non- availability of KMC providers	Social obligation -fulfilling gender roles	Associating LBWI birth to a spiritual punishment and a consequence of diversion norm		Lack of women empowerment in decision making	Compromised quality care -poor documentation, monitoring and follow-up
		Learning about KMC when after giving birth to an LBWI	LBWI identified as ‘these kinds of people’			Inadequate skill by KMC provider

### The trustworthiness of the results

Issues of credibility, transferability, dependability and conformability to ensure trustworthiness of the study findings were as follows: credibility; incorporated research methodologies similar to the concept under study, voluntary participation that facilitated getting honest information from the participants, use of probing questions to ignite detailed information and triangulation of findings from FGDs and observational data collection to verify some details. Transferability; the study results understood within the context of PLBWIs, which ensured transferability to other settings by using this study’s methods. Dependability; the study was executed according to the study protocol, to obtain reliable study findings. Conformability; the study findings are a result of the triangulation of results from FGDs and the observational findings, which ensured the conformability of the results.

The mean (± standard deviation [SD]) age for enrolled women was 32.2 (±8.7) years.

### Characteristics of the study population

The mean (± standard deviation [SD]) age of the 12 participants was 23.3 (±8.2) years. Out of 12 participants, 8.3% (1) were not married and 91.7% (11) were married. Of 12 participants, 83.3% (10) attended primary school, 8.3% (1) did not go through formal education and 1 (8.3%) completed secondary education. There was an association between level of education and employment, such that 91.7% (11) of the participants whose education was below primary school were unemployed unlike 8.3% (1) who completed secondary school. Of the 12 participants, 58.3% (7) had been pregnant before while 41.7% (5) was their first time being pregnant. Of the seven mothers who had been pregnant before, 85.7% (6) had had three pregnancies and more. Of the 12 participants, high blood pressure, HIV/primigravida/adolescent. Sexual Transmitted Infection and twin gestation denoted 8.3% (1) each of the predisposing factor to LBWI delivery; while HIV had 33.3% (4) and primigavida/adolescent had 33.3% (4).

The mean (± standard deviation [SD]) gestational age of the seven LBWIs was 34.2 (±1.5) weeks. The mean (± standard deviation [SD]) birthweight of the seven LBWIs was 1958.3 (±441.3) grams. Out of the seven LBWIs 57.1% (4) were female while 42.9% (3) were male. Primigravida merged the highest attributing factor to preterm delivery with 57.1% (4) then twin gestation at 28.5% (2). 85.7% (6) of the LBWIs delivered preterm while 14.3% (1) were small for gestational age.

### Availability of KMC service

Two sub-themes emerged from this factor namely; availability and non-availability of KMC providers, as presented below.

The checklist on the availability of KMC service (see Additional file 3) at MDH showed that the essential equipment for KMC service and KMC providers were available. The KMC provider confidently assessed the LBWIs and skillfully provided KMC service. This concurred with the narrative of a mother who delivered at this hospital.

*“When I delivered, I was told to put the baby on my chest. They said I should put the baby on my chest, which helps that when my heart beats it will remind my baby that outside the womb there is a life of breathing and also the baby should not be exposed to cold to avoid the baby’s body to become cold. As such, it should be kept warm all the time because if the baby is exposed to cold it can die at any time” PLBWI 4.*

KMC service was also available in other distant facilities, which referred the clients to MDH. The mother who delivered at a distant health facility and referred to MDH for further management explained the availability of KMC at her delivery facility.

*“At Katuli health centre they said they don't have the machine to put the baby on, so they said I should just keep it on a kangaroo. That time the baby was grunting, in the morning we embarked on an ambulance to come here. When we came here the grunting stopped and we were taught how to put the bay on the chest, its advantages and its disadvantages”. PLBWI 1*

Some of the distant health centres did not have a residing trained/skilled KMC provider as experienced by these mothers;

*“I heard about kangaroo at the hospital because I was lucky during one of the antenatal visits I met the visiting nurse, she gave kangaroo education. She said, “when you are pregnant expect that you can give birth before or after you complete your months. If you give birth at home, do not just sit back but go to the hospital the baby is put on kangaroo”. I was lucky to have received the education because she visited the hospital on the day of my appointment. The nurses at the hospital did not know about kangaroo, the visiting nurse who was coming and delivering the kangaroo education at antenatal” PLBWI 4.*

*“I carried my baby on my laps from Phirilongwe to here; the nurse did not explain anything. She just said you will find the assistance right there in Mangochi, and she did not say the kind of care I was going to get. Here, they took my baby straight to an electrical room. My baby stayed there for two days thereafter I started kangaroo” PLBWI 3.*

*“I gave birth at Nangalamu…they sent me here because the baby was born before its time, and they do not do kangaroo. The only care I got on this baby is that the nurse wrapped my baby in a blanket and placed it in my arms and told me to come here and I carried it in my arms to here” PLBWI 6.*

### Accessibility of KMC service

Health linkage system, knowledge of KMC, social support and encouragement, and social obligations were the sub-themes that emerged as factors that facilitated the accessibility and utilization of KMC service.

PLBWIs perceived linkages between health centres and MDH for further management as a facilitating factor for accessing KMC services.

*“They said the care that my baby will be getting would be inadequate; hence, they sent me here at Mangochi to get better care” PLBWI 4.*

Knowledge of KMC considered as one of the factors facilitating access to KMC services, although not all mothers had prior knowledge of KMC. The source of KMC knowledge services varied, from friends, the media and health workers. Some mothers only heard of KMC services when they had given birth to an LBWI.

*“I knew about kangaroo at home, a long time ago from people who gave birth to a baby born before its time. She was doing kangaroo” PLBWI 2.*

*“… I also heard it from the radio that giving birth to a low birth weight baby is not something strange. The doctors have ways to help you” HRPM 5.*

*“At the antenatal what we were told is****,****if a pregnant woman has signs of malaria-like fever, she should go to the hospital as soon as possible because, in the long run, she might give birth to a baby born before its due date. If she has body pains, you have to go to the hospital to address your complaint so that you should be helped and give birth at the right time to a mature baby” PLBWI 1.*

Despite the disparities in the timing of KMC knowledge, the mothers narrated the advantages of KMC as described below:

*“The goodness of kangaroo that I have seen, the way the baby was, I did not know that it can survive. When the baby was born, I could not see the lips and the ears well. Now I can see the ears and the lips. I can also touch them” PLBWI 2.*

*"My babies were not crying neither were they moving, but when I came to the hospital the babies started moving, crying and opening their eyes. Because of these, I believe that Kangaroo mother care can help people if they can seek for help quickly".PLBWI 3.*

*“Am happy the baby is now receiving enough care, by putting the baby on the chest” PLBWI 1.*

*“…a baby born before its time and put on kangaroo mother care to me is not yet a child and putting the child on kangaroo gives me hope that one day my child will become a real child and I will say I have given birth to a mature child because of the kangaroo” PLBWI. 4*

Only a few participants acknowledged having support social support and encouragement with KMC practice, the majority did not have any kind of support as narrated by some mothers:

*“Yes, I do kangaroo the whole day I have someone who helps me. She is apparently outside” PLBWI 5.*

*“I do not do kangaroo all the time. I also have twins and I have one person who supports me. Therefore, when I want to go to the bathing room, wash the nappies I put the baby on the bed” PLBWI 3.*

### Acceptability of KMC service

Recognition of LBWIs, social factor; maternal affection, safety on the use of KMC, preference of LBWI’s care practice, lived experience and use of KMC expert clients, perceived causes of.

BWI births, cultural factors; religious and traditional beliefs were the sub-themes, which emerged under KMC service acceptability.

All the participants accepted the LBWIs and displayed maternal affection towards their LBWIs despite them not recognizing them [LBWIs] as not-yet babies.

*“It is God wishes for us to have these kinds of babies. As such, this is what God has given us as such we accept them” PLBWI 3.*

*“I cannot throw the baby away maybe it can survive and help me someday. The baby can turn up to be either president, teacher or something else important” HRPM 3.*

Most participants perceived KMC as safe to use, while other mothers found the use of KMC as a death trap to the infants.

*“I do not see any danger in putting the baby on kangaroo mother care provided I look to it that I put the baby nicely that I should not pin any of its organs” PLBWI 3.*

*“The baby will be deprived of air when it is in her mother’s clothes” HRPM 3.*

KMC, incubator care and traditional care of an LBWI emerged as care practices for LBWIs.

Some participants preferred KMC to incubator care, while many other participants preferred incubator care to KMC; while yet others practised traditional ways of caring for LBWIs.

*“There is love between a mother and her mature born baby, but eeeh this one is number one putting the baby on the chest makes the mother love the baby more, as it has come before its time” PLBWI 3.*

*“I also prefer electricity care, I would not mind if the baby stays in that care for months provided the baby gets better” HRPM 2.*

*“Our forefathers believed that when a baby has come before her days, it had to be wrapped in blankets and placed on the bed with a hot charcoal stove underneath it, for warm. It is our belief up to today” PLBWI 2.*

The majority of participants had a positive lived experience with KMC and promised to be KMC expert clients,^1^ while few participants had a bad lived experience with the practice.

*“I will encourage the mothers who will give birth before the babies time to do kangaroo. The decision should be theirs. I can tell them that you see my babies came before their time, I was helped by the nurses and I did kangaroo and but now see my babies are healthy. Unlike them staying at home, they cannot gain anything”. PLBWI 2.*

*"We can encourage them saying the way things are, do kangaroo. Others will be adamant because they had practised, and the babies died. Those who have doubts in kangaroo, they have said it is better to cover the baby in blankets at home than doing kangaroo". HRPM 1*

The participants associated LBWI delivery to having sexually transmitted infections antenatal due to promiscuity. Religious and traditional beliefs included as the causes of LBWI delivery. So do the intentional abortion, which was associated with cultural taboo. These brought about stigma and affected the acceptability of the LBWIs and KMC service.

*“Some people say, for a woman to give birth to a baby born before its time it means that when the woman was pregnant the husband was sleeping with other women and he brought sexual infections in the home. Hence, it caused the woman to give birth early”. PLBWI 1*

*“People ask why you gave birth to a baby before its time/ and they talk bad things. They say you were ill talking the babies born before the actual time, so the spirits have punished you”. PLBWI 3*

*“The act of just sleeping with other women when his wife is pregnant causes the wife to delivery before the baby is due”. PLBWI 6.*

*“People say mockery words, saying she tried to abort the baby and now see her small baby. Others would come to see the baby on kangaroo and talk behind your back saying “have you see the child?” The baby is small and looks like a mouse one cannot even see its head. As such, when a kangaroo mother passes by a group of women, they start gossiping about you. Then, she will say its better I stay at home and I do not go anywhere” PLBWI 6.*

### Affordability of KMC service

Most participants viewed KMC as expensive care to access compared to those who perceived it as affordable.

*“Kangaroo is not involving because hospital process is different from that of the traditional healer. At the traditional healer, one can spend a lot of money than at the hospital, and not healed” HRPM 5.*

*“I do not worry about the expenses provided the baby gets better, unlike getting worried about the expenses and ending up destroying human life” PLBWI 1.*

### Personal behavior

Health seeking behavior and women empowerment were the sub-themes that emerged under personal behavior.

All the participants expressed a zeal to seek health attention.

*"I will agree to practice kangaroo mother care because I want a baby. Getting pregnant and stay for ten months and God gives me that [referring to an LBWI], so when God gives me and the doctors tell me what to do and if there was something that I was doing I would leave all that to concentrate on the future of the baby". HRPM 3*

Most of the mothers depended on their husbands to authorize KMC utilization, while some depended on their mothers-in-law, who had an upper hand in decision-making. Only a few mothers had a shared responsibility with their husbands in decision-making.

*“The nurse told me that my babies were not matured yet, so there is a need to take them to kangaroo. I just said ok fine. Then, I sent a message at home that here I have given births to babies that are not mature, so they should come over to help me with KMC…I also told my husband.” PLBWI 4*

*“I can call my husband to get authorization, whether he is in Johannesburg or at the lake fishing telling him that this is what has happened to me and the doctor says I should be in the hospital for two months… I will still wait until I speak to him”. HRPM 4.*

### Quality of care

Sub-themes identified under quality care include skills of KMC providers and quality of care.

During the assessment of KMC availability, the study found out feeding charts, treatment charts and KMC register were not consistently charted and updated. Although, either a nurse or a student nurse staffed the KMC unit 24 h, the unit did not have a reporting book to record and report the progress of the LBWIs at the daily hospital-morning report sessions.

Additionally, observed that the student nurses lacked the necessary KMC skills and confidence to counsel the PLBWI, to the point that the PLBWI seemed not interested with KMC counselling.

## Discussion

The study aimed to assess the facilitating factors and barriers to accessibility and utilization of KMC service by PLBWIs. In this study, the accessibility of KMC service described as availability, accessibility, acceptability, affordability of KMC service, personal behavior and quality of care, and utilization of KMC service was referred to the utilization of the service.

### Availability of KMC service

This study found that the availability of providers at the secondary and most of the primary level of health service delivery in Mangochi district promoted utilization of KMC service. Although that was the case, some health centers did not have KMC providers, which hindered pregnant mothers to access KMC information at antenatal care (ANC) and utilization of the service at postnatal. This coincides with the study finding that revealed that availability of material and human resources for the implementation of KMC at any level of service provision, facilitate the utilization of the service [2, 25, 26]. Inadequate skills of student nurses in KMC counselling and service provision contributed to the non-acceptance and poor utilization of KMC service. The study results concur with the WHO recommendation on the availability of a trained and skilled KMC provider for the accessibility and utilization of the service [25]. Tasking shifting was not the case at MDH where KMC unit not staffed 24 h a day with skilled and trained KMC provider due to inadequate of skilled nurse providers to cover KMC unit throughout the day and the presumed workload associated with KMC service, which hindered access and utilization of KMC by the beneficiaries. This finding agrees with other studies that ascertain that KMC providers’ availability throughout the day in cases of health workers experiencing workload, task shifting is exercised whereby patient’s attendants are trained to provide KMC service to ensure continuous availability and utilization of KMC [3, 27].

### Accessibility of KMC service

This study found out that consistency and compliance of KMC practice was possible to some mothers who had family and social support. Our study findings coincided with other studies which show that family support enhances the mother to practice KMC 24 h, which promotes KMC accessibility and utilization [28, 29]. Mothers who had challenges with family support and needed to fulfil gender roles did not practice KMC throughout the day, which compromised accessibility and utilization of KMC. The finding is similar to this study that concedes mothers who have poor family support system have challenges in practicing KMC 24 h [30].

Some mothers had KMC awareness through their peers, antenatal and media and they accessed and accepted KMC utilization in time when they gave birth to an LBWI. Chisenga et al.*,* concur that prior knowledge of KMC intervention and its efficiency enhances its accessibility, acceptability and use when the need arises [31]**.** However, some mothers did not have prior knowledge of KMC intervention from all avenues of KMC knowledge dissemination, including at ANC visits that identified as an important avenue to disseminate KMC messages to pregnant women and their spouses. Despite HIV, adolescent pregnancies and increased number of pregnancies been the high risk factors of LBWIs deliveries in most of the PLBWI in this study; and LBWIs births and preterm deliveries that require KMC service, pregnant mothers were deprived of KMC messages at ANC. The non-dissemination of KMC messages at ANC affected acceptability and utilization of KMC on timing. Messages/guidelines in the Malawi antenatal counselling standard operating procedures (SOPs) does not integrate KMC awareness at the ANC service delivery point [32, 33], which is aiding inconsistency in KMC message acquisition amongst mothers at ANC clinics in various health facilities, although,. Lydon et al.*,* observed that the ANC clinic was the important arena to disseminate KMC messages to the targeted population, who are at risk of LBWI delivery [34]. This study indicated increased number of pregnancies as one of the high risk factors of LBWIs deliveries in MD catchment area, which serves as the indicators to dissemination of KMC messages at ANC clinics and strengthening strategies of family planning methods uptake. WHO denotes that access and utilization of Family Planning (FP) services is essential in preventing unplanned pregnancies, hence indirectly preventing preterm delivers [35]. Regardless of the mothers’ knowledge on KMC, mothers who delivered LBWIs at the health centres who needed further management, including KMC, were referred to a secondary level facility for inpatient KMC service, which facilitated accessibility and utilization of KMC. A Malawi study narrates that strong referral linkage systems have proven to maximize the accessibility and utilization of quality KMC services [34].

### Acceptability of KMC service

Cultural, religious and traditional beliefs such as the association of LBWI delivery to being punished by the spirits and committing a cultural taboo of abortion subjected mothers to ridicule and hindered the recognition of LBWIs and subsequently the non-acceptability of KMC service and its utilization. Studies done in Ghana and South Africa concur that cultural, traditional and religious beliefs on the causes of LBWIs’ delivery affect the perception towards LBWIs and consequently prevent KMC acceptability and utilization [18, 19]. Despite LBWIs, been considered as not yet human beings and practicing KMC was a cause of ridicule; mothers in this study accepted LBWIs and utilized KMC due to their maternal love towards their LBWIs. Feldman et al. correspond with this study’s finding that parental affection towards LBWIs enables the parents to accept the LBWIs and utilize KMC for the betterment of the LBWI [19]. Although mothers utilized KMC, some mothers preferred incubator care and traditional way of caring for the LBWIs to KMC for fear of subjected to ridicule. The studies done in Ghana, Malawi and Mali add that mothers who accepted their LBWIs and practiced KMC were considered cultural norm diverters and they were discriminated and ridiculed, which compromised the utilization of KMC service [36–38].

Some mothers considered KMC as not a safe intervention to use for the LBWIs as they considered it as a death trap for LBWIs, this compromised consistency of KMC utilization. The result is similar to the findings of a study in Malawi and a twenty-nice included a systematic review that found out that some mothers felt unsafe when using KMC than incubator care [6, 28]. This substantiated with evidence of bad experience that some mothers witnessed a baby dying whilst on KMC position, which brought uneasiness in some mothers when utilizing KMC. This is similar to a finding in Bergh et al*’s.,* study that previous unpleasant outcome with KMC deters the acceptability of KMC. Nonetheless, some mothers preferred KMC due to its capability of maternal-infant bonding, affection and safety that promoted acceptability and utilization. This finding concurs with the study done by Chisenga et al.*,* in which perceived KMC advantages facilitated acceptability of KMC service and the WHO declaration of no dangers associated with KMC use promotes KMC acceptability and utilization [31, 39]. The mothers who experienced the advantages of KMC had positive experience towards KMC, which prompted them to pledge to act as expert clients by encouraging others faced with a similar situation to practice KMC, which aided in acceptability and utilization of KMC. Expert clients are known in playing a role in motivating mothers to utilize KMC [40], therefore, these KMC expert clients might influence KMC accessibility, acceptability and utilization.

### Affordability of KMC service

Most of the PLBWIs, in this study, were unemployed. Even though Malawi offers free health services, including KMC service, some mothers perceived KMC as expensive due to its demand for a long stay in the hospital and the extra demand for material and financial resources associated with the service, which may deter KMC utilization. Unemployment of the PLBWIs did not affect the utilization of KMC, as KMC service was free of charge. Lipato clarifies that there is a long stay in the hospital for the unstable LBWIs this is due to a need to stabilise the infants before KMC is initiated [41]. Although studies found that KMC reduces hospital stay for LBWIs as compared to LBWIs on conventional care [42].

### Personal behavior

This study revealed that mothers who portrayed a positive personal behavior towards LBWIs demonstrated health-seeking behavior in utilizing KMC service, which is similar to the finding of a qualitative systematic review of 29 studies that parents who had a positive personal behavior in KMC utilized the service [28]. Nonetheless, some studies found personal health behavior negatively affected by the age, number of pregnancies of the mother and primigravida in adolescent mothers [43], which was not the case in this study that denoted the high prevalence of LBWI deliveries in adolescent mothers. The adolescent mothers had the zeal to practice KMC. Asides health behavior, this study indicated that mothers who had authority in decision making accepted KMC utilization and initiated KMC in time, which is similar to the finding of a systematic review that found that couples that equally contributed to making decision utilized KMC service [28]. This was not the case with some mothers who portrayed gender inequality and lack of women empowerment in decision making to access KMC service, such that gender roles played an influential role in decision-making. Women who had less or no authority in accepting and utilizing KMC without the node from their husbands either delayed in KMC initiation and/or did not accept the service. An MDG 4 review study and Chisenga et al.*,* coincide with the finding of this study that gender roles influence decision-making in seeking health service [35], such that most of the mothers depend on their husbands to decide on practicing KMC [31]. Therefore, gender inequality and lack of women empowerment prevent mothers from making health decision on their own, which hinders seeking health services in time, which has an impact in increased incidences of preventable neonatal deaths [2, 35].

### Quality of care

In this study, monitoring and follow-up of the LBWIs and tracking the progress of the LBWIs on KMC services was not consistently done, which affected the organization of the unit and the parental zeal to utilize KMC. Follow-up and monitoring of care help to ascertain the quality of care and the impact of KMC service on LBWIs mortality and morbidity. This finding is similar to what Smith et al.*,* find that quality of the health service has a major influence on health-seeking behavior and the compromised quality of health services facilitates the underutilization of the service [35]. Asides poor monitoring and follow-up, the study found that inadequate skill by the KMC provider in KMC counselling discouraged mothers from accepting and utilizing KMC service. Other study found out that inadequacy of trained health workers contributes to the compromised quality of the health service [44].

## Conclusion

The impact of KMC service on LBWIs mortality depends not only on the implementation of the services but also on the understanding of facilitating factors and barriers encountered by the users of KMC service, that will inform on the strategies to be employed to address the issues that deter KMC accessibility and utilization. The key findings on facilitating factors included the availability of service providers, family support, dissemination of KMC messages at ANC, referral linkages, perceived KMC advantages and safety and women empowerment in decision-making. The key challenges included lack and inadequate of skilled service providers, lack of family support, non-integration of KMC messages at ANC, lack of women empowerment in decision-making. Although, in this study, timing of KMC knowledge acquisition did not affect KMC utilization among the women who had preterm delivery and LBWIs, but non-dissemination of KMC message at the ANC clinic was a missed opportunity to disseminate KMC message to the novice mothers and those that had never heard about KMC. In this study, most of the pregnant women were primigravidae and adolescent, nonetheless, the adolescent pregnancy did not affect the utilization of KMC. Therefore, integration of KMC messages in antenatal care guidelines, community awareness and sensitization of any health intervention may enhance KMC accessibility and utilization by the targeted population (adolescent, pregnant women and those who had had increased number of pregnancies), in turn preventing LBWIs’ mortality. Further studies to be conducted to identify recommended strategies to be employed to sensitize the community with KMC messages, to enhance KMC awareness among the targeted population.

## Supplementary information

**Additional file 1.** Focus Group Discussion (FGD) Guide: Parents of Low Birth Weight Infants (LBWIs).

**Additional file 2.** Focus Group Discussion (FGD) Guide: High-risk pregnant women.

**Additional file 3.** Kangaroo Mother Care (KMC) Availability Checklist For Researcher to Assess KMC Unit Availability.

## Data Availability

Data from this study are the property of the Government of Malawi and University of KwaZulu-Natal and cannot be made publicly available. All interested readers can access the data set from Malawi’s National Health Sciences Research Committee (MNHRSRC) and the University of KwaZulu-Natal Biomedical Research Ethics Committee (BREC) from the following contacts: THE CHAIRMAN, MINISTRY OF HEALTH (RESEARCH DEPARTMENT), P.O Box 300377, Lilongwe 3, Tel: (+ 265) 6017 26422, Fax: (+ 265) 017 26418, Email: cmwansambo@malawi.net or rmajamanda@gmail.com. The Chairperson BIOMEDICAL RESEARCH ETHICS ADMINISTRATION Research Office, Westville Campus, Govan Mbeki Building University of KwaZulu-Natal P/Bag X54001, Durban, 4000 KwaZulu-Natal, South Africa Tel.: + 27 31 260 4769 Fax: + 27 31 260 4609 Email: BREC@ukzn.ac.za

## Abbreviations

ANC: Antenatal Care
BREC: Biomedical Research Ethics Committee
FGS: Focused Group Session
HRPM: High-risk pregnant mother
KMC: Kangaroo Mother Care
LBWIs: Low Birth Weight Infant
MDH: Mangochi District Hospital
NHSRC: National Health Sciences Research Committee
PLBWI: Parent of Low Birth Weight Infant
SDG: Sustainable Development Goals
STIs: Sexually Transmitted Infections
UKZN: University of KwaZulu-Natal
WHO: World Health Organization

## Footnotes

1 Clients who have undergone a KMC experience and deliberately use their lived experience to help others faced with similar situations to mitigate challenges [24]
