# Supplementary material for: Facilitating factors and barriers to accessibility and utilization of kangaroo mother care service among parents of low birth weight infants in Mangochi District, Malawi: a qualitative study
Source: BMC Pediatr. 2020 Jul 29;20:355. doi: 10.1186/s12887-020-02251-1 (PMC7390197; doi:10.1186/s12887-020-02251-1)
Supplement: Supplementary file 1 — Additional file 1. Focus Group Discussion (FGD) Guide: Parents of Low Birth Weight Infants (LBWIs). [file 12887_2020_2251_MOESM1_ESM.docx]

## Additional file 1: Focus Group Discussion (FGD) Guide: Parents of Low Birth Weight Infants (LBWIs)

**Background**

Low Birth Weight (LBW) is the leading cause of neonatal deaths globally as well as locally. The Malawi Ministry of Health (MoH) implements Kangaroo Mother Care (KMC) to manage LBWIs, among other neonatal health initiatives.

KMC is a skin-to-skin technique between the caregivers and the Low Birth Weight Infants’ (LBWIs), which has proven a success in preventing 50% of the LBWIs’ deaths. The success of KMC utilization lies on availability, accessibility, affordability, acceptability and health seeking behaviour of individuals.

This FGD is part of the researcher’s academic project, which aims at identifying and describing the barriers, challenges and facilitating factors to KMC utilization by parents of LBWIs

**Purpose of the focus group discussion**

The purpose of the focus group discussion is to explore:

- How Low Birth Weight Infants (LBWIs) are viewed
- Alternative ways of managing LBWIs in the community
- Parents of LBWIs’ knowledge on KMC service
- Parents of LBWIs’ perception on KMC service
- How acceptable is KMC to their community
- What are parents of LBWIs’ concerns about KMC
- The parents of LBWIs’ opinions on the challenges, barriers and facilitating factors to the utilisation of KMC
- The parents of LBWIs’ recommendations on improvement of KMC utilization

**General Information**

| District: |  |
| --- | --- |
| **District zone:** |  |
| **Data collection:** |  |
| **Date of data collection:** |  |

**Discussion Guide**

1. Where do most of the pregnant women deliver? And why?

**Probe:**

1. Where do the least of the pregnant women delivery? And why?
2. What were your expected outcomes of pregnancy?

**Probe:**

1. Was LBWI one of the pregnancy outcomes?
2. During antenatal visits, were you taught about LBWI being one of the outcomes of pregnancy?
3. How are the LBWIs viewed in your community? And why?
4. What happens when the woman gives birth to a LBWI at the place where the majority of the pregnant women give birth?

**Probe:**

1. What happens when the woman gives birth to a LBWI at the place where the least of the pregnant women give birth? And why?
2. Where did you gave birth? And what happened?
3. What are the dangers that can occur to a LBWI?
4. In your community how are LBWIs managed?
5. What interventions does the health facility provide for a LBWIs?

**Probe:**

1. Which intervention do you prefer? And why?
2. When did you learn about KMC?
3. Were you taught about KMC during your antenatal clinics?

**Probe:**

- 1. What do you understand about KMC and LBWIs? And why?

1. *What do you think are the advantages of KMC?*
2. *What do you think of KMC safety to a LBWI*?
   1. How do you feel about KMC?
3. *Is there any change in your child since you started using KMC service? Explain*
4. *Are you going to continue KMC practice at home? And why?*
5. *Are there any cultural/religious beliefs associated with LBWIs and KMC? Explain*
   1. What does your community say about KMC and LBWIs? And why?
6. *How do your community view a caregiver who practice KMC? And why?*
7. *How is/will your community view you as you practice KMC? And why? (Acceptability)*
8. *Is KMC acceptable in your culture?*
   1. What is your opinion on KMC services? And why?
9. *Do you utilize KMC service throughout the day? And why? (availability/accessibility)*
10. *Is KMC costly? And why? (affordable)*
11. *How long did it tool you to start practising KMC? And why? (accessibility/availability)*
12. When you gave birth who authorised that the LBWI be managed on KMC? And why?

**Probe:**

1. How long did it take for the decision maker to authorise for KMC?
2. What influenced the decision for KMC? Explain
   - 1. *What issues were taken into consideration in making a decision? (Distance, transport, cost, outcome of the LBWI, health seeking behaviour?)*
3. What do you think are the challenges, barriers and facilitating factors to the utilisation of KMC?
4. What do you recommend to be done in order to facilitate KMC utilization? And why?
5. Can you recommend KMC? And Why?
