# Supplementary material for: Facilitating factors and barriers to accessibility and utilization of kangaroo mother care service among parents of low birth weight infants in Mangochi District, Malawi: a qualitative study
Source: BMC Pediatr. 2020 Jul 29;20:355. doi: 10.1186/s12887-020-02251-1 (PMC7390197; doi:10.1186/s12887-020-02251-1)
Supplement: Supplementary file 2 — Additional file 2. Focus Group Discussion (FGD) Guide: High-risk pregnant women. [file 12887_2020_2251_MOESM2_ESM.docx]

## Additional file 2: Focus Group Discussion (FGD) Guide: High-risk pregnant women

**Background**

Low Birth Weight (LBW) is the leading cause of neonatal deaths globally as well as locally. The Malawi Ministry of Health (MoH) implements Kangaroo Mother Care (KMC) to manage LBWIs, among other neonatal health initiatives.

KMC is a skin-to-skin technique between the caregivers and the Low Birth Weight Infants’ (LBWIs), which has proven a success in preventing 50% of the LBWIs’ deaths. The success of KMC utilization lies on availability, accessibility, affordability, acceptability and health seeking behaviour of individuals.

This FGD is part of the researcher’s academic project, which aims at identifying and describing the barriers, challenges and facilitating factors to KMC utilization by parents of LBWIs

**Purpose of the focus group discussion**

The purpose of the focus group discussion is to explore:

- How Low Birth Weight Infants (LBWIs) are viewed
- Alternative ways of managing LBWIs in the community
- Pregnant women’s knowledge on KMC service
- Pregnant women’s perception on KMC service
- How acceptable is KMC to their community
- What are pregnant women’s concerns about KMC
- The pregnant women’s’ opinions on the challenges, barriers and facilitating factors to the utilisation of KMC
- The pregnant women’s recommendations on improvement of KMC utilization

**General Information**

| District: |  |
| --- | --- |
| **District zone:** |  |
| **Data collection:** |  |
| **Date of data collection:** |  |

**Discussion Guide**

1. Where do most of the pregnant women deliver? And why?

**Probe:**

1. Where do the least of the pregnant women delivery? And why?

2. What are your expected outcomes of pregnancy?

**Probe:**

- 1. Is LBWI one of the pregnancy outcomes?
  2. During your antenatal visits, have you been you taught about LBWI being one of the outcomes of pregnancy?

3. How are the LBWIs viewed in your community? And why?

4. What happens when the woman gives birth to a LBWI at the place where the majority of the pregnant women give birth?

**Probe:**

- 1. What happens when the woman gives birth to a LBWI at the place where the least of the pregnant women give birth? And why?
  2. Where are going to give birth? Why?

1. What are the dangers that can occur to a LBWI?
2. In your community how are LBWIs managed?
3. During your antenatal visit have you been taught about the interventions that the health facility provides for a LBWI? Explain

**Probe:**

- - 1. Which intervention do you prefer? And why?

1. Have you been taught about KMC?

**Probe:**

- 1. What do you understand about KMC and LBWIs? And why?
     1. what do you think are the advantages of KMC?
     2. What do you think of KMC safety to a LBWI?
  2. How do you feel about KMC?
     1. *Do you think KMC is worthy doing? And why?*
     2. *If you happen to give birth to the LBWI, are you going to use KMC service? And why?*
     3. *Are you going to continue KMC practice at home? And why?*
  3. What does your community say about KMC and LBWIs? And why?
     1. *How do your community view a caregiver who practice KMC? And why?*
     2. *How will your community view you as you practice KMC? And why? (Acceptability)*
     3. *Is KMC acceptable in your culture?*
  4. What is your opinion on KMC services? And why?

1. *Do you think you will be able to utilize KMC service throughout the day? And why? (availability/accessibility)*
2. *Do to think KMC is costly? And why? (affordable)*
3. *When you give birth to a LBWI, how long do you think it will take you to start practising KMC? And why? (accessibility/availability)*
4. If you happen to give birth to a LBWI, who has the authority to authorise that the LBWI to be managed on KMC? And why?

**Probe**

1. How long do you think it can take for the authorization of KMC?
2. What do you think can influence the decision for KMC? Explain
3. *What issues can be taken into consideration in making a decision? (Distance, transport, cost, outcome of the LBWI, health seeking behaviour?)*
4. What do you think are the challenges, barriers and facilitating factors to the utilisation of KMC?
5. What do you recommend to be done in order to facilitate KMC utilization? And why?
6. Can you recommend KMC? And Why?
