# Supplementary material for: Facilitating factors and barriers to accessibility and utilization of kangaroo mother care service among parents of low birth weight infants in Mangochi District, Malawi: a qualitative study
Source: BMC Pediatr. 2020 Jul 29;20:355. doi: 10.1186/s12887-020-02251-1 (PMC7390197; doi:10.1186/s12887-020-02251-1)
Supplement: Supplementary file 3 — Additional file 3. Kangaroo Mother Care (KMC) Availability Checklist For Researcher to Assess KMC Unit Availability. [file 12887_2020_2251_MOESM3_ESM.docx]

# **Additional file 3: Kangaroo Mother Care (KMC) Availability Checklist**

**For Researcher to Assess KMC Unit Availability**

**Name of District: _________________________________________________**

**Name of Health Facility: ___________________________________________**

**No. Health Providers supervised by cadre:**

1. **Clinicians____________________________________________________**

1. **Nurses______________________________________________________**
2. **Others (specify) ______________________________________________________**

**______________________________________________________**

**Name of Researcher(S): ____________________________________________**

**Date of Supervision: ______________________________________________**

**Instructions**

Use this checklist to assess KMC unit/ward. The checklist itemizes all the areas that should be supervised. In filling in this checklist, the researcher or is expected to physically check or observe the activity/item being supervised. Under checklist column, tick **(X) as follows;**

- For an activity/ item that has been perfectly done, tick under 3
- For an activity/item not done tick under 2
- For an activity/item that you failed to observe during the supervision, tick under 1.
- The comments column is for any additional information you may have regarding how the activity/item was done. Write under 4
- The researcher is expected to supervise **ALL** the activities/items

| **Area** | **Activity/Item observed** | **1** | **2** | **3** | **4** |
| --- | --- | --- | --- | --- | --- |
|  |  | **Not observed** | **Not done** | **Done** | Comment on the quality of care |
| 1. Service provision | Initial Counselling |  |  |  |  |
|  | Routine education |  |  |  |  |
|  | Discharge talk |  |  |  |  |
|  | Assisting/coaching mothers on KMC positioning |  |  |  |  |
|  | Coaching for breast feeding a LBW baby |  |  |  |  |
|  | Baby’s vital signs are checked and documented |  |  |  |  |
|  | Monitoring of feeds |  |  |  |  |
|  | Monitoring of weight gain |  |  |  |  |
| 1. Staff coverage | Nursing ward round done at least once daily |  |  |  |  |
|  | 24-hour coverage by a nurse or nurse auxiliary/patient attendant in the KMC unit. |  |  |  |  |
|  | Clinical ward rounds at least three times a week |  |  |  |  |
| 1. Documentation | Information in KMC register or any other recording books is updated and properly documented |  |  |  |  |
|  | All discharged babies are appropriately recorded in the follow up forms. |  |  |  |  |
| 1. Inclusion in the routine report discussions | Any LBW baby and care (KMC) is reported in the daily morning report sessions |  |  |  |  |
|  | LBW/KMC data is part of HMIS reports and discussions |  |  |  |  |
| 1. Supplies and Equipment | Availability of appropriate weighing scale (*electronic or digital baby scale with 1g to 20g analogues)* |  |  |  |  |
|  | Availability of feeding cups |  |  |  |  |
|  | Availability of appropriate NGTs *(size 6 and 8)* |  |  |  |  |
|  | Availability of IP utensils and solutions  *(buckets with chlorine solution)* |  |  |  |  |
| 1. Conduciveness of the KMC unit | Availability of head adjustable beds or beds with pillows |  |  |  |  |
|  | Availability of Running water for hand washing |  |  |  |  |
|  | The room is well illuminated |  |  |  |  |
|  | Clean room |  |  |  |  |
|  | Windows are closable |  |  |  |  |
|  | Toilets are within easy reach |  |  |  |  |
|  | Bath rooms are within easy reach |  |  |  |  |
| 1. Follow up system | Followed up in the KMC unit |  |  |  |  |
|  | Facility keeps traceable physical address of the babies |  |  |  |  |
|  | Inventory of HSAs with their catchments area |  |  |  |  |
| 1. Integration of KMC into normal MCH/maternity | Percentage of LBW/premature babies referred to KMC unit documented |  |  |  |  |
| 1. IEC materials and management protocols | KMC leaflets for mothers |  |  |  |  |
|  | KMC eligibility criteria |  |  |  |  |
|  | List of danger signs |  |  |  |  |
|  | Feeding chart |  |  |  |  |
|  | KMC posters |  |  |  |  |

**Final Comments/ observation:** *Based on your observation, state how the KMC service providers are working in terms of knowledge of KMC, confidence, counselling skills, rapport/attitude with parents of low Birth Weight Infants (LBWIs)*

_______________________________________________________________________________________________________________________________________________________________________________________________________________________________________________________________________________________________________________________________________________________________________________________________________________________________________________________________________________________________________________________________________________________________________________________________________________________________________
